# Rapid Eye Movement (REM) Sleep Behaviour Disorder in Moebius Syndrome: A Rare Pediatric Case

**DOI:** 10.7759/cureus.101029

**Published:** 2026-01-07

**Authors:** Cláudia P Gonçalves, Ana Sofia Nunes, Augusta Gonçalves, Marta Ribeiro Silva, Carla Moreira

**Affiliations:** 1 Pediatrics, Unidade Local de Saúde de Braga, Braga, PRT; 2 Pediatric Pneumology, Unidade Local de Saúde de Braga, Braga, PRT

**Keywords:** moebius syndrome, parasomnias, pediatric sleep disorders, rem sleep behaviour disorder, sleep medicine

## Abstract

Moebius syndrome is a rare, non-progressive congenital disorder, most commonly characterized by impairment of the abducens (VI) and facial (VII) cranial nerves, resulting in facial palsy and limited ocular abduction. A broad spectrum of associated clinical manifestations includes craniofacial abnormalities, limb malformations, and neuromotor developmental delay. Sleep disorders are frequently reported in these patients, most often related to sleep-disordered breathing. In contrast, rapid eye movement (REM) sleep behavior disorder (RBD) is exceptionally rare, both in pediatric patients and in association with this syndrome.

We report the case of a 13-year-old male diagnosed with Moebius syndrome in the neonatal period, who developed recurrent episodes of nocturnal agitation, vocalizations, and dream enactment behaviors. Polysomnography demonstrated structural alterations of REM sleep with reduced muscle atonia and abnormal motor activity, findings consistent with RBD. Despite the implementation of sleep hygiene measures and pharmacological therapy, clinical response was limited.

This case underscores the importance of actively investigating sleep disturbances in Moebius syndrome, not only to address the more common sleep-related breathing disorders but also to recognize rare conditions, such as RBD, which may significantly impact quality of life. Given the scarcity of evidence on pediatric RBD, particularly in association with congenital neurological syndromes, further research is needed to improve diagnostic and therapeutic strategies.

## Introduction

Moebius syndrome is a rare, non-progressive congenital disorder first described in 1880. Although its etiology remains poorly understood, mutations in genes such as PLXND1 and REV3L have been associated with autosomal dominant forms of the condition [1]. It is characterized by unilateral or bilateral paresis of the seventh cranial nerve (facial nerve) and is frequently associated with impaired ocular abduction due to involvement of the sixth cranial nerve (abducens nerve) [2-4]. Other cranial nerves, such as the glossopharyngeal (IX) and hypoglossal (XII) nerves, may also be involved [1,5]. In addition to neurological abnormalities, craniofacial dysmorphisms [1] and skeletal limb malformations, such as clubfoot and brachydactyly, are common [2,4]. Hypoplasia of the pectoralis major muscle, known as Poland anomaly, is also frequently observed in these patients [1,4].

Regarding psychomotor development, motor developmental delay is common, and cognitive and behavioral alterations may also be present [2,4], which may have a significant impact on daily functioning and academic performance, particularly during childhood and adolescence.

Sleep disturbances are common in patients with Moebius syndrome, with obstructive sleep apnea syndrome being frequently diagnosed [4]. Although previously described, rapid eye movement (REM) sleep behavior disorder (RBD) is rare in this population [6].

REM RBD is uncommon in pediatric patients overall and is characterized by dream-enactment behaviors during REM sleep, including vocalizations and complex or violent motor activity, resulting from the loss of the physiological muscle atonia that normally characterizes this sleep stage [7]. These episodes may lead to self-injurious conduct or aggression toward others [8]. In children and adolescents, RBD has been most frequently reported in association with neurodevelopmental disorders, brainstem abnormalities, and narcolepsy type 1 [7].

Importantly, sleep quality and sleep architecture play a critical role in emotional regulation, learning, and cognitive function. Sleep disturbances, including sleep fragmentation and recurrent nocturnal motor and behavioral events, may contribute to daytime consequences, such as impaired attention, behavioral dysregulation, emotional lability, and reduced school performance, in adolescents [9]. Within this spectrum of sleep disturbances, disorders affecting REM sleep may have a particularly relevant clinical impact due to their association with complex nocturnal behaviors and sleep fragmentation.

In this context, the occurrence of RBD in a patient diagnosed with Moebius syndrome is of particular clinical interest, as it may reflect underlying brainstem involvement in sleep regulation. Case reports of this nature can contribute to improved recognition of sleep disturbances in children and adolescents with congenital neurological disorders, allowing for earlier diagnosis, optimized clinical management, and improved quality of life.

## Case presentation

A 13-year-old male adolescent, born in Brazil and living in Portugal for the past two years, was diagnosed with Moebius syndrome in the neonatal period. The diagnosis was established based on clinical findings, including congenital facial involvement manifested by absent facial expression, bilateral congenital clubfoot, and orofacial involvement with tongue hypotonia. At birth, he was admitted to a neonatal intensive care unit, requiring ventilatory support with non-invasive ventilation (nCPAP). Family medical history was unremarkable.

Regarding the patient’s personal medical history, particular attention was given to growth and neurodevelopmental evolution. In terms of growth parameters, weight progressed from the 3rd-10th percentile during the first 4 months of life to the 50th-85th percentile thereafter, while length remained consistently between the 3rd and 10th percentiles since birth. Concerning neurodevelopment, there was a history of global developmental delay, with delayed independent walking and significant language delay. Neurodevelopmental assessment using the Wechsler Intelligence Scale for Children (WISC-III) indicated intellectual disability, and in the school setting, the patient requires special and adapted educational support.

Due to the multisystemic nature of Moebius syndrome and the need for ongoing multidisciplinary follow-up, he was referred by his primary care physician to General Pediatrics, Orthopedics, Physical Medicine and Rehabilitation, Ophthalmology, Pediatric Cardiology, Otolaryngology, and Pediatric Pulmonology appointments.

At the Pulmonology consultation, caregivers reported restless sleep associated with screaming and crying, as well as episodes of somniloquy and bruxism. Further sleep history revealed an average of nine hours of sleep per night, with snoring and excessive sweating. There were no reported breathing pauses, nocturnal enuresis, headaches, or daytime sleepiness. There was no history of sleepwalking. The physical examination was notable for overweight (body mass index between the 85th and 97th percentiles), low-set ears, micrognathia, and tonsillar hypertrophy grade III/IV according to the Brodsky scale.

**Figure 1:**
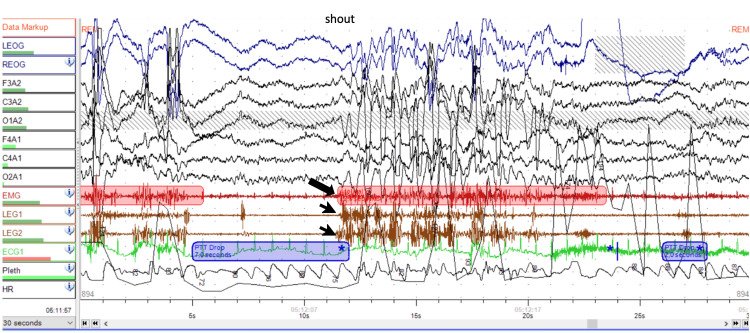
Nocturnal polysomnography performed with a 32-channel recording system, including six electroencephalographic (EEG) leads, electrooculogram (EOG), chin electromyogram (EMG), and bilateral lower limb EMG The recording shows a period of REM sleep, during which a vocalization (shout) is observed, temporally associated with loss of chin muscle atonia (arrow) and increased phasic muscle activity in the lower limbs (arrowheads), consistent with REM sleep behavior disorder.

Given these complaints, a polysomnography was performed (Figure 1). Periods identified as REM sleep showed an absence of ocular movements (predominantly horizontal), increased motor activity, and reduced chin muscle atonia. REM sleep accounted for 13.5% of total sleep time (a reduced percentage), during which two episodes of agitation and screaming were recorded. These structural sleep alterations, associated with episodes of agitation, were suggestive of REM sleep behavior disorder. In addition, respiratory events compatible with upper airway resistance syndrome were identified: Apnea-Hypopnea Index (AHI) of 1.1 and Respiratory Disturbance Index (RDI) of 7.4.

In collaboration with Otolaryngology, and after a lack of improvement in snoring with medical therapy using nasal corticosteroids and montelukast, surgical treatment was performed via partial tonsillectomy and inferior turbinectomy, which resulted in an initial improvement followed by symptom recurrence.

Regarding RBD, sleep hygiene measures were implemented, and clonazepam was initiated before bedtime, with progressive dose titration, without significant improvement in the episodes. During follow-up, there was worsening of nocturnal agitation, including self-injurious behavior.

The patient remains under multidisciplinary follow-up and continues treatment with clonazepam and nasal corticosteroid therapy, with no satisfactory clinical response.

## Discussion

Moebius syndrome is a rare condition with a markedly heterogeneous clinical presentation, most commonly characterized by the involvement of the abducens (VI) and facial (VII) cranial nerves [4], resulting in unilateral or bilateral facial palsy and oculomotor deficits, particularly impaired ocular abduction [1]. Due to the wide variability of its clinical features, the establishment of definitive diagnostic criteria for this syndrome has proven challenging, making the assessment and follow-up of these patients a complex and often multidisciplinary task [1].

Since hypotonia and craniofacial anomalies, such as micrognathia, are common features in Moebius syndrome [4], it is expected that these patients may present with sleep-disordered breathing. In the present case, in addition to micrognathia, the presence of grade III tonsillar hypertrophy and excess body weight represent additional factors contributing to the observed snoring and, consequently, to the upper airway resistance syndrome identified on polysomnography. Management of sleep-disordered breathing should be individualized, taking into account symptom severity, physical examination findings, and polysomnographic results [9]. Available therapeutic strategies include weight loss, medical treatment with nasal corticosteroids and/or montelukast, and surgical intervention through tonsillectomy; in severe cases, continuous positive airway pressure (CPAP) therapy should be considered [9].

Although the prevalence of sleep-disordered breathing is high in the pediatric population, RBD is rare in this age group. REM sleep is characterized by vivid dreams associated with near-complete muscle atonia, sparing only the diaphragm and extraocular muscles, and plays an important role in the maturation of neural circuits [10]. In RBD, vivid dreams are often accompanied by vocalizations and body movements--sometimes potentially injurious--resulting from the loss of the physiological muscle atonia that characterizes this sleep stage [8].

In pediatric patients, RBD occurs primarily in association with type 1 narcolepsy, developmental delay, or brainstem abnormalities [9]. Given that the Moebius syndrome is a congenital disorder with predominant involvement of brainstem structures and cranial nerve nuclei, the occurrence of RBD in this context is biologically plausible [6]. Although uncommon, RBD has been reported in patients with Moebius syndrome, as illustrated by the present case.

Regarding therapeutic options, the management of RBD in children remains challenging; clonazepam is the most widely used pharmacological agent, although its efficacy is variable and not always satisfactory [5].

## Conclusions

Sleep disorders, particularly sleep-disordered breathing and REM sleep disturbances, should be actively investigated in patients diagnosed with Moebius syndrome. Furthermore, research on REM sleep behaviour disorder in the pediatric population, especially regarding therapeutic approaches, should remain a priority in order to optimize follow-up and improve the quality of life of these patients.
